# Supplementary material for: Transcriptomic Insights into Mechanisms of Early Seed Maturation in the Garden Pea (Pisum sativum L.)
Source: Cells. 2020 Mar 23;9(3):779. doi: 10.3390/cells9030779 (PMC7140803; doi:10.3390/cells9030779)

Zhewan−1,10(2)

Global Summary

%DE = 0.24

genes with fdr < 0.2 = 466 (212 + / 254 −)

genes with fdr < 0.1 = 391 (186 + / 205 −)

genes with fdr < 0.05 = 358 (168 + / 190 −)

genes with fdr < 0.01 = 326 (152 + / 174 −)

genes in genesets = 0

<FC> = 0

<t−score> = 0

<p−value> = 0.01

<fdr> = 0.76

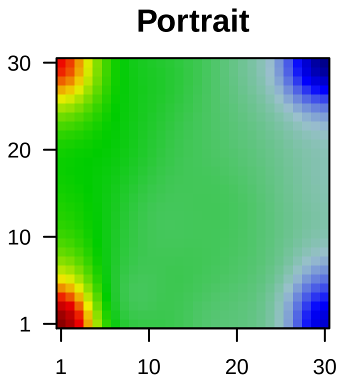

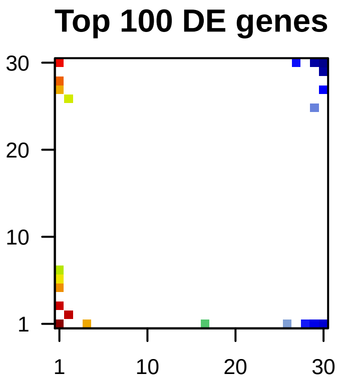

Global Genelist

Rank	ID	log(FC)	p−value	fdr	Metagene	Description
1	TRINITY_DN1	−0.66	2e−16	3e−15	30 x 1	
2	TRINITY_DN1	0.79	2e−16	3e−15	1 x 3	
3	TRINITY_DN9	−0.54	2e−16	3e−15	30 x 30	
4	TRINITY_DN7	−2.46	2e−16	3e−15	30 x 1	
5	TRINITY_DN7	2.05	2e−16	3e−15	30 x 30	
6	TRINITY_DN5	−2.39	2e−16	3e−15	30 x 30	
7	TRINITY_DN5	1.78	2e−16	3e−15	1 x 1	
8	TRINITY_DN5	1.53	2e−16	3e−15	1 x 30	
9	TRINITY_DN7	−0.63	2e−16	3e−15	30 x 30	
10	TRINITY_DN7	1.85	2e−16	3e−15	1 x 1	
11	TRINITY_DN9	−0.62	2e−16	3e−15	30 x 30	
12	TRINITY_DN9	−0.98	2e−16	3e−15	30 x 30	
13	TRINITY_DN9	−0.55	2e−16	3e−15	30 x 1	
14	TRINITY_DN2	0.88	2e−16	3e−15	1 x 30	
15	TRINITY_DN2	0.66	2e−16	3e−15	1 x 1	
16	TRINITY_DN2	−0.75	2e−16	3e−15	30 x 1	
17	TRINITY_DN2	−0.53	2e−16	3e−15	30 x 1	
18	TRINITY_DN2	−1.26	2e−16	3e−15	30 x 30	
19	TRINITY_DN2	1.12	2e−16	3e−15	1 x 30	
20	TRINITY_DN2	1.33	2e−16	3e−15	1 x 1	

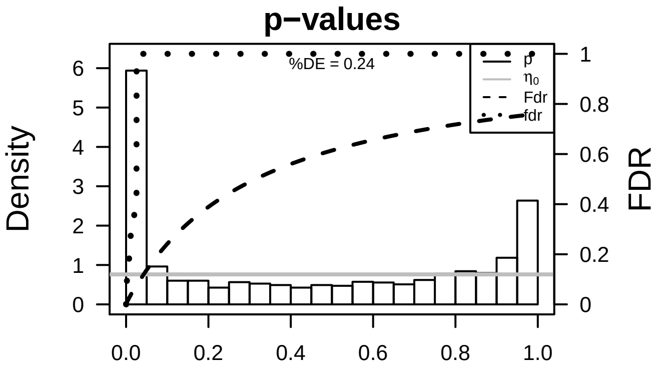

Supplement: Supplementary file 1 [file cells-09-00779-s001.zip › Supplementary materials/FileS4.zip!/FileS3/TFs - Results/Summary Sheets - Samples/Zhewan.1.10.2..pdf]
